# Online Social Networking Sites and Mental Health Research

**DOI:** 10.3389/fpsyt.2015.00036

**Published:** 2015-03-12

**Authors:** Umar Toseeb, Becky Inkster

**Affiliations:** ^1^School of Psychological Sciences, University of Manchester, Manchester, UK; ^2^Department of Psychiatry, Wolfson College, University of Cambridge, Cambridge, UK

**Keywords:** Facebook, social networking sites, mental health, psychiatry, friendship, depression

Socializing and networking was transformed in the technological era by the introduction of social networking sites (SNSs). These online sites contain an abundance of information about individual preferences, interests, types, and frequency of social interactions, etc. However, scientific studies that have utilized SNS activity data to aid our understanding of mental health disorders are scarce. This is partly due to the practicalities of accessing SNS data and methodological issues of large-scale data collection, but also because the construct validity of SNS measures is unknown. By and large, the literature to date has attempted to link various SNSs measures to various mental health symptomologies, mostly collected using self-report measures rather than data generated by SNSs. Although such research has demonstrated some preliminary and putative associations between SNS activity and mental health measures, the current literature is still in its infancy and arguably lacks rigor in design, offering limited insights into its theoretical significance and plausibility. In this review, we will provide an account of the theoretical importance of using data generated from SNSs in mental health research and provide a brief overview of the literature published in this area to date.

## Introduction

The world of socializing and networking was reinvented in the technological era by the introduction of online SNSs and other forms of digital social media such as MySpace, Bebo, Hi5, Facebook, Twitter, YouTube, Google, Instagram, and Vine. The shift into the digital online environment has left social networking users with digital footprints that generate a relatively unique set of identifiers, both in online and offline worlds.

In 2012, Facebook (one such SNS) reached a staggering one billion monthly users (1) meaning that approximately 1/7th of the world’s population were regular Facebook users. Usage is especially high among young people. Livingstone et al. (2) reported that 26% of 9- to 10-year-olds, 49% of 11- to 12-year-olds, 73% of 13- to 14-year-olds, and 82% of 15- to 16-year-olds have their own profile on an SNS. Moreover, 51% of 13- to 18-year-olds log on to their online social networking profile at least once a day, 34% log on more than once a day, and 22% check it more than 10 times per day (3). These figures provide insight into the extremely popular online culture of SNSs, especially among young people.

## Why Study Social Networking Sites in Mental Health Research?

The extraordinary popularity of online SNSs does not alone warrant their use for research purposes; therefore, it is important to understand what SNSs add to existing methodology in the field of mental health research. SNS activity logs leave behind a digital trail of quantifiable and objective data that can be arguably valuable to researchers. It may be thought of as analogous or complementary to observing individuals in their natural environment more so than conventional self-report measures of present and past behaviors, which may result in reporting bias in adolescents (4). Such biases could be in the form of reporting distorted behaviors, which are in fact demand characteristics, which may be less problematic in large datasets and also in digital trails of SNSs. As such, creating and maintaining friendships, interactions with friends, cyber-bullying, specific interests, etc., could potentially be assessed more directly and with greater accuracy and precision using online information. Issues surrounding self-report methods such as test–retest reliability may be overcome by using online activity logs. Some SNSs enable users to have a virtual existence with personal sociodemographic details (e.g., name, age, current, and previous towns/countries of residence all available to view). Such advances in social networking and internet technology offer mental health researchers new tools and opportunities for large-scale data collection, analysis, and interpretation that were previously not possible.

Several studies have shown evidence that social network profiles convey fairly accurate personality portrayals rather than idealized virtual identities of profile owners (5). Most typically, friendships are formed in an offline-to-online sequence; peer-reviewed statements about their friend’s offline interests and values support the accuracy of their online identities [reviewed by Wilson et al. (6)]. While arguably some profile users might engage in self-enhancement and narcissistic self-promotion, research has shown that independent raters can accurately detect such profiles as narcissistic behaviors (7). Facebook recently reported that 8.7% of Facebook user profiles were “fake” (8). However, only ~1.5% were actually defined as “undesirable profiles” (i.e., profiles that breached Facebook terms and conditions). The remaining fake profiles included such things as duplicate profiles for business and organization purposes or to create non-human profiles (e.g., for family pets). The 1.5% “undesirable profiles” are generally used to send spam messages or corrupted content to other FB users. Although this percentage of undesirable fake profiles is low, one possible way to minimize this occurrence is to establish study recruitment procedures that are initially offline and then acquire the verified Facebook users online profile data.

The sheer scale of SNSs popularity can be considered as a strong scientific asset as it provides a high dimensional and dense log of behavioral information, which can enhance the power to detect small effects of complex behaviors associated with mental illness. SNSs can capture additional and unique information of how people lead their lives, offline and online. Both sets of experiences could be very similar for some individuals but very different for others. It may well be that data collected from SNSs is a different method of measuring the same behaviors; alternatively, a whole range of new behaviors could be observed. Previous technological advances such as the television, music players, and games consoles have all been very passive in their nature and it was difficult to derive data from them, whereas SNSs require some level of participation from the user. Moreover, it may not be enough to simply look at online data in general (e.g., Google searches) because families often share a computer and so individual differences may not be detected. Online SNSs allow for this “statistical noise” in the data be reduced and allow one-to-one mapping of individuals online and offline behavior. The interaction between online and offline behaviors may also be of interest to researchers and provide unique insights into understanding mental health. Another advantage of measuring data from SNSs is the rapid and dense collection of data within extraordinarily smaller timescales that are highly cost-effective [e.g., methodology used by Kosinski et al. (9)].

Given that a large proportion of lifetime mental health problems develop in adolescence and young adulthood (10), early intervention and prevention that are targeted at young people would provide personal and economic benefits. Early detection and assessment of mental illnesses would help to reduce poorer life outcomes (10). Early intervention can have a significant impact on those who experience mental health problems, whether this come from discoveries of early biomarkers or emerging deviations in behaviors. Emerging research indicates that intervening early can interrupt the negative course of some mental illnesses and may, in some cases, lessen long-term disability. Changes in online behaviors may offer novel insights and naturalistic measures, “red flags” to inform prevention and early detection strategies.

## What Work has been Done so Far?

Although there has been some limited and preliminary work on the relationship between SNSs and mental health, there appears to be a scarcity of mental health literature that extracts large-scale data from SNSs in an attempt to better understand mental health disorders. Literature does exist that employs self-report questionnaires to gather data about SNS usage. This is important because it provides rationale for future research using data generated by SNSs. SNSs have the potential to produce data on hundreds of thousands, if not millions of people from different parts of the world. Although such large datasets have the potential to produce spurious associations [e.g., (11)], these can be dealt with using hypothesis-driven statistical analysis. Furthermore, SNSs usage data can be easily shared with other researchers and so would move forward the drive to share datasets in an effort to replicate research and reduce the number of false positives.

Using relatively small sample sizes (*n* > 500) and self-report methods of Facebook use, some research studies have found that various parameters on Facebook (e.g., friend count, social support, and time spent on Facebook) related to depressive symptoms or well-being (12–15); although this is not entirely supported (16). Researchers have also found that various Facebook parameters such as status updates were able to predict depressive symptoms (17–19). Additionally, Good et al. (20) found that looking back over old posts and photos on a user’s own profile had a positive effect on well-being and that reminiscing had more of a positive effect on well-being for those who had mental health problems in the past compared to those with no previous mental health problems. More recently, Frison and Eggermont (21) found that passive Facebook use (consuming other peoples information without interacting with them) was associated with depressed mood in girls and active public Facebook use (interacting with other friends such that the interactions are visible to others, e.g., status updates) was associated with depressed mood in boys.

Other than the predominantly cross-sectional studies, a limited number of research studies use stronger research designs such as longitudinal and experimental. Longitudinal studies have an advantage over cross-sectional work in that they allow for an exploration into change over time, causality, and the association between variables at different time points. For example, Kross et al. (22), over a period of 2 weeks, investigated the relationship between Facebook use and subjective well-being five times a day. They found that increased use of Facebook at one time-point predicted lower well-being at the next time point. Also by text messaging participants, Verduyn et al. (23) found that passive Facebook use (consuming information from Facebook without interacting with other users) was associated with lower well-being. Furthermore, an experimental study by Sagioglou and Greitemeyer (24) asked participants about their well-being immediately after Facebook use and found that longer use predicted lower mood. This finding is particularly relevant as it implies causality as Facebook use was measured directly prior to well-being.

There is considerable variability in the quality of the work that has been produced using self-report methods of SNS usage and mental health. One of the main reasons for this is due to the lack of validity in some of the reported measures. For example, Burke et al. (25) found that there was a significant correlation between self-report friend count and actual friend count (*r* = 0.96) and also between self-report time spent on Facebook and actual time spent on Facebook (*r* = 0.45). Junco (26) also investigated the difference between actual Facebook use (monitored by computer monitoring software) and self-reported use. Although time spent on Facebook was correlated for the two measures (*r* = 0.6), there was a significant difference between them. That is, participants overestimated the time spent on Facebook (mean difference = 123 min per day). It may be that self-report is a more reliable measure for simple measures such as friend count but when looking at more complex measures it becomes less reliable.

Very few studies have taken a computational approach to utilizing Facebook parameters. Kosinski et al. (9) used the Facebook “like” feature, which allows users to specify what he/she likes or has an interest in (e.g., type of music, music bands, movies, interests, past times, places, etc.). Participants consented for researchers to extract data about their profile that is automatically collected by Facebook. These researchers found that what someone “likes” on Facebook can be used to predict, with a relatively high degree of accuracy, his/her sexual orientation (0.88), race (0.95), and voting preferences (0.85). There are only a few studies in mental health research, which have used more complex online behavior traits generated by SNS data. For example, Burke et al. (25) and Burke et al. (27) discuss the concept of social support and how it can be measured through Facebook behaviors such as the type and frequency of the content produced and consumed. This is important because increased social support has been linked to a decrease in depressive symptoms [e.g., Brown et al. (28)]. An interesting example of online data (not generated by SNSs) is given by Ayers et al. (29) who found that Google searches for certain mental health disorders varied by the seasons of the year.

It is not possible to reach any conclusions based on the limited amount of literature in relation to mental health research. However, this is a very important area to examine in much greater detail. Lessons can be learned from Facebook research in psychological and social science contexts and used in designs for mental health research.

## Summary

Online SNSs are increasingly popular in people’s everyday lives and as they leave behind a cumulative digital trail of activity data they should be of considerable interest to mental health researchers. There is a growing body of literature around the association between behaviors on SNSs and mental health, but research that uses activity history on SNSs and how that links to mental health is scarce. This activity log of information is important because it has the potential to provide researchers with large amounts of data, which is not only easy to obtain but less dependent on a research funding as the data collection costs are vastly smaller. The problem with these large datasets is that self-report studies that should inform hypotheses and research questions are often poorly designed. Moreover, due to the novelty of the data collection method, there are unanswered questions about its construct validity and what research using this method can add to theoretical constructs in the field.

## Conflict of Interest Statement

The authors declare that the research was conducted in the absence of any commercial or financial relationships that could be construed as a potential conflict of interest.
